# SAMHD1 impairs type I interferon induction through the MAVS, IKKε, and IRF7 signaling axis during viral infection

**DOI:** 10.1016/j.jbc.2023.104925

**Published:** 2023-06-14

**Authors:** Constanza E. Espada, Levent Sari, Michael P. Cahill, Hua Yang, Stacia Phillips, Nicholas Martinez, Adam D. Kenney, Jacob S. Yount, Yong Xiong, Milo M. Lin, Li Wu

**Affiliations:** 1 Department of Microbiology and Immunology, Carver College of Medicine, University of Iowa, Iowa City, Iowa, USA; 2 Lyda Hill Department of Bioinformatics, University of Texas Southwestern Medical Center, Dallas, Texas, USA; 3 Department of Molecular Biophysics and Biochemistry, Yale University, New Haven, Connecticut, USA; 4 Department of Microbial Infection and Immunity, The Ohio State University, Columbus, Ohio, USA

**Keywords:** SAMHD1, HIV-1, innate immunity, interferon, IRF, MAVS, IKKε, molecular docking

## Abstract

Sterile alpha motif and HD domain-containing protein 1 (SAMHD1) restricts human immunodeficiency virus type 1 (HIV-1) infection by reducing the intracellular dNTP pool. We have shown that SAMHD1 suppresses nuclear factor kappa-B activation and type I interferon (IFN-I) induction by viral infection and inflammatory stimuli. However, the mechanism by which SAMHD1 inhibits IFN-I remains unclear. Here, we show that SAMHD1 inhibits IFN-I activation induced by the mitochondrial antiviral-signaling protein (MAVS). SAMHD1 interacted with MAVS and suppressed MAVS aggregation in response to Sendai virus infection in human monocytic THP-1 cells. This resulted in increased phosphorylation of TANK binding kinase 1 (TBK1), inhibitor of nuclear factor kappa-B kinase epsilon (IKKε), and IFN regulatory factor 3 (IRF3). SAMHD1 suppressed IFN-I activation induced by IKKε and prevented IRF7 binding to the kinase domain of IKKε. We found that SAMHD1 interaction with the inhibitory domain (ID) of IRF7 (IRF7-ID) was necessary and sufficient for SAMHD1 suppression of IRF7-mediated IFN-I activation in HEK293T cells. Computational docking and molecular dynamics simulations revealed possible binding sites between IRF7-ID and full-length SAMHD1. Individual substitution of F411, E416, or V460 in IRF7-ID significantly reduced IRF7 transactivation activity and SAMHD1 binding. Furthermore, we investigated the role of SAMHD1 inhibition of IRF7-mediated IFN-I induction during HIV-1 infection. We found that THP-1 cells lacking IRF7 expression had reduced HIV-1 infection and viral transcription compared to control cells, indicating a positive role of IRF7 in HIV-1 infection. Our findings suggest that SAMHD1 suppresses IFN-I induction through the MAVS, IKKε, and IRF7 signaling axis.

Sterile alpha motif and HD domain-containing protein 1 (SAMHD1) is an enzyme with deoxynucleotide triphosphohydrolase (dNTPase) activity. The ability to regulate the pool of cytosolic dNTPs allows SAMHD1 to restrict replication of viruses that depend on cellular dNTPs for genome replication, such as DNA viruses (1, 2, 3) and retroviruses (4, 5). Of note, SAMHD1 restricts human immunodeficiency virus type 1 (HIV-1) in noncycling cells, such as macrophages, dendritic cells, and resting CD4+ T cells (6, 7, 8, 9). However, SAMHD1 mutations that reduce the dNTP pool without restricting HIV-1 infection have been described, suggesting a dNTPase-independent HIV-1 restriction mechanism (10, 11). Germline mutations in the *SAMHD1* gene are associated with development of Aicardi-Goutières syndrome, an autoimmune disease characterized by a type I interferon (IFN-I) dysregulation (12, 13, 14). Furthermore, spontaneous induction of IFN-I in SAMHD1-deficient human monocytes has been reported (15), suggesting a physiological role of SAMHD1 in regulating the IFN-I pathway.

We have demonstrated that SAMHD1 suppresses nuclear factor kappa-B and IFN-I signaling pathways in response to pro-inflammatory stimuli and virus infections (16, 17, 18). SAMHD1 interacts with inhibitor of nuclear factor kappa-B kinase epsilon (IKKε) and IFN regulatory factor 7 (IRF7) leading to inhibition of phosphorylation of IRF7 by IKKε. Moreover, SAMHD1 inhibited the activity of an IFN-sensitive response element (ISRE) reporter induced by IRF7, but not IRF3 overexpression (16). IFN-I are involved in limiting the replication of pathogens (19, 20), modulating immune cell homeostasis and function (21) and activating the adaptive immune response (22). Thus, understanding the mechanism by which SAMHD1 antagonizes IFN-I is of crucial importance.

The IFN-I response in virus-infected cells establishes an antiviral state in neighboring cells to limit virus spread (23). Upon virus infection, pathogen-associated molecular patterns, such as viral dsRNA, are recognized by the cytoplasmic sensors retinoic acid–inducible gene I (RIG-I) and melanoma differentiation-associated gene 5 (MDA-5) (24). Signal propagation occurs through a common adapter protein called mitochondrial antiviral-signaling protein (MAVS) (25, 26, 27). Upon engagement of pathogen-associated molecular patterns, conformational changes in RIG-I and MDA-5 facilitate activation of MAVS by phosphorylation and polymerization (28, 29, 30) followed by recruitment and activation of IKK-related kinases, TBK1, and IKKε (31, 32). These kinases phosphorylate IRF3/7 transcription factors, leading to their dimerization and nuclear translocation where they promote IFN-α/β transcription (33, 34). In most cell types, IRF3 is constitutively expressed, providing a fast antiviral response through the transcription of *IFN-β* and certain *IFN-α* genes. In contrast, IRF7 is highly induced by IFNs and activates the transcription of a larger set of *IFN-α* genes, leading to amplification of the IFN-α/β response (35).

In this study, we provide new insights into the molecular mechanisms by which SAMHD1 antagonizes IFN-I induction signaling. Our results show the capacity of SAMHD1 to suppress IFN-I responses relies on its ability to directly interact with key proteins in the RIG-I–like receptors (RLR) pathway, such as MAVS, IKKε, and IRF7. We show that SAMHD1 interacts with MAVS and inhibits MAVS aggregation and activation. SAMHD1 interaction with IKKε disrupts IRF7 binding to IKKε, which explains the ability of SAMHD1 to block IRF7 phosphorylation by IKKε. We demonstrate that SAMHD1 suppression of the IRF7-mediated antiviral response depends on its interaction with the inhibitory domain of IRF7 (IRF7-ID). We also identify three residues in IRF7-ID, essential for transactivation activity and SAMHD1 binding. Furthermore, we showed that IRF7 is required for efficient HIV-1 infection and viral transcription in THP-1 cells. Our findings revealed new mechanisms by which SAMHD1 suppresses IFN-I induction through the MAVS, IKKε, and IRF7 signaling axis in the context of viral infection, which help better understand the role of SAMHD1 in innate immunity.

## Results

### SAMHD1 inhibits MAVS-mediated IFN-I signaling and interacts with MAVS

**Figure 1:**
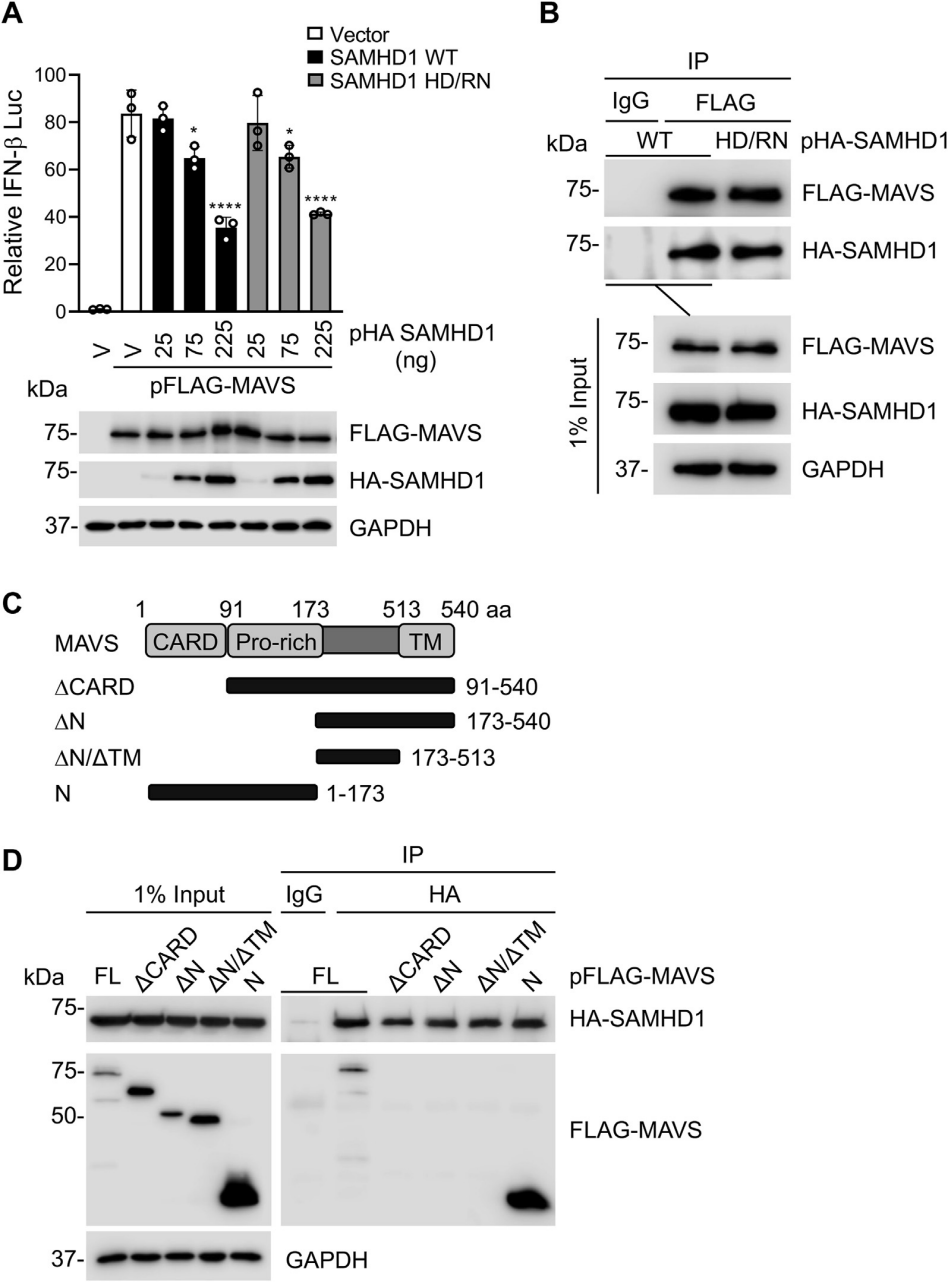
**SAMHD1 inhibits MAVS-mediated IFN-I signaling and interacts with the CARD of MAVS.***A*, HEK293T cells were cotransfected with increasing amounts of plasmid encoding HA-SAMHD1 WT or HD/RN, IFN-β-luciferase reporter, renilla-TK, and FLAG-tagged MAVS. Dual luciferase assay was performed at 24 h posttransfection, and cell lysates were harvested for IB. Error bars represent mean ± SD. Statistical significance was determined using one-way ANOVA; ∗∗*p* < 0.01; ∗∗∗∗*p* < 0.0001 compared with the vector control in the same group. *B*, HEK293T cells were cotransfected with plasmids encoding HA-SAMHD1 WT or HD/RN and FLAG-MAVS. Cells were lysed and IP was performed using anti-FLAG antibody at 36 h posttransfection. Nonspecific IgG was used as a negative control in IP, and the indicated proteins were detected by IB. *C*, schematic representation of full-length (FL) MAVS and MAVS mutants. The amino acid (aa) numbers of MAVS are shown. The CARD, Pro-rich, and TM domains are indicated. *D*, HEK293T cells were cotransfected with plasmids encoding HA-SAMHD1 and FLAG-MAVS or MAVS mutants. Cells were lysed and IP was performed using anti-HA antibody at 36 h posttransfection. Nonspecific IgG was used as a negative control in IP, and the indicated proteins were detected by IB. *A*, *B* and *D*, representative data from three independent experiments are shown. CARD, caspase-recruitment domain; HD, histidine-aspartate; IB, immunoblot; IFN, interferon; MAVS, mitochondrial antiviral-signaling protein; Pro-rich, proline-rich; SAMHD, sterile alpha motif and HD domain-containing protein; TM, transmembrane; V, vector controls.

We have reported that SAMHD1 inhibits IFN-I signaling induced by the overexpression of IRF7, but not IRF3 (16). To better understand the underlying mechanisms, we first asked whether SAMHD1 inhibits IFN-I signaling upstream of IRF7 by components of the RLR pathway. HEK293T cells were cotransfected to express a luciferase reporter under control of the IFN-β promoter, MAVS, and increasing amounts of SAMHD1 WT or a dNTPase-defective HD/RN mutant (H206R and D207N) (36). Overexpression of SAMHD1 WT or HD/RN inhibited MAVS-induced IFN-β promoter activity in a dose-dependent manner (Fig. 1*A*). The highest level of SAMHD1 WT or HD/RN expression suppressed MAVS-induced IFN-β luciferase reporter by approximately 50% (Fig. 1*A*), suggesting that SAMHD1 suppresses MAVS-mediated IFN-I signaling independently of its dNTPase activity.

We then utilized co-immunoprecipitation (Co-IP) to determine whether SAMHD1 interacts with MAVS. We found that SAMHD1 WT and HD/RN similarly interacted with full-length (FL) MAVS (Fig. 1*B*), indicating that the binding is independent of the dNTPase activity of SAMHD1. MAVS contains three functional domains, including the caspase-recruitment domain (CARD), a proline-rich region (Pro-rich), and a transmembrane domain (TM) (28). To identify the domain of MAVS required for the interaction with SAMHD1, we generated four truncation mutants of MAVS (Fig. 1*C*). HEK293T cells were cotransfected with plasmids expressing WT SAMHD1 and FL MAVS or individual MAVS mutants (Fig. 1*C*). WT SAMHD1 co-immunoprecipitated with FL MAVS; however, deletion of the CARD of MAVS (ΔCARD) or two MAVS mutants lacking the CARD and Pro-rich domains (ΔN and ΔN/ΔTM) abrogated their interactions with SAMHD1 (Fig. 1*D*). We then generated and tested the MAVS mutant (N) expressing CARD and Pro-rich domains, which efficiently bound to WT SAMHD1 (Fig. 1*D*). Together, these data suggest that SAMHD1 interacts with the CARD of MAVS.

### SAMHD1 localizes to the mitochondria and directly interacts with MAVS

**Figure 2:**
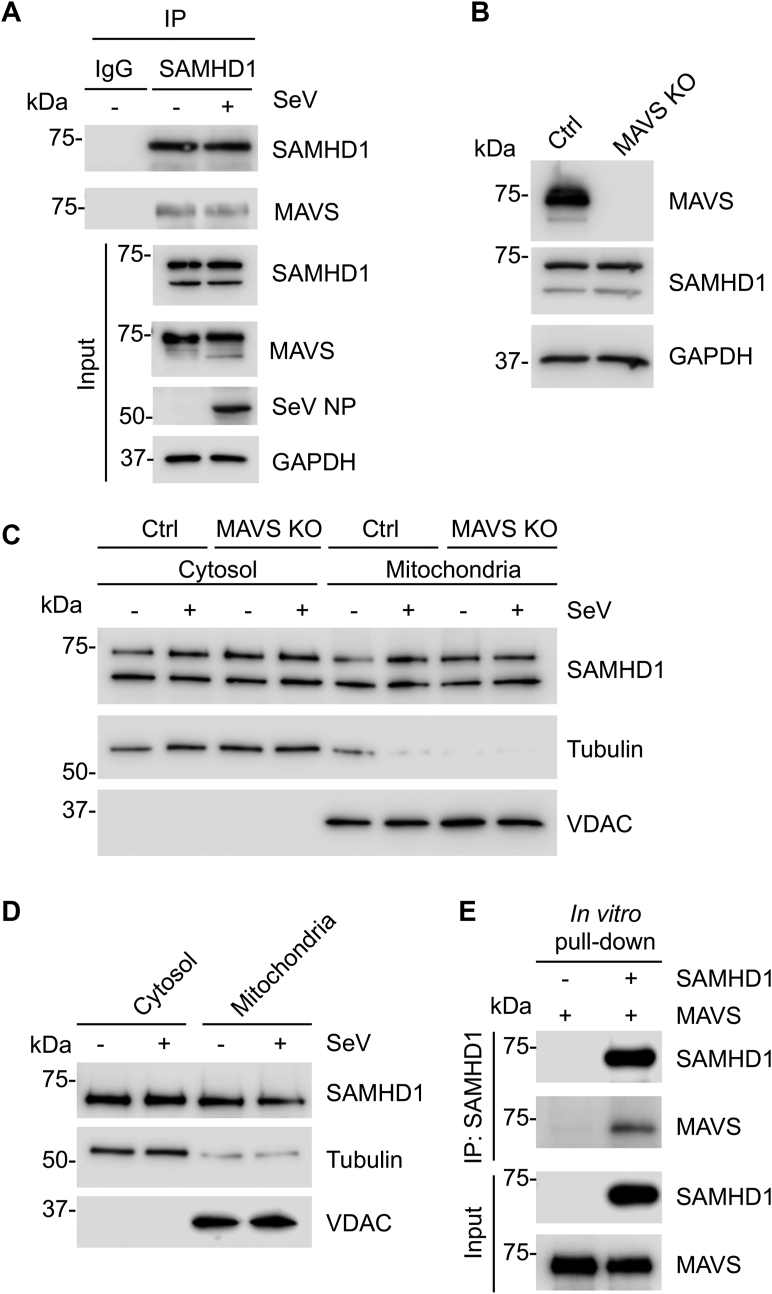
**SAMHD1 localizes to the mitochondria and directly interacts with MAVS.***A*, THP-1 control cells were infected with SeV (MOI of 10) and harvested at 6 hpi for IP with SAMHD1 antibody or IgG control. IB was performed to detect the indicated proteins. Two bands of SAMHD1 in input samples represent the splicing variants. *B*, IB analysis of MAVS and SAMHD1 expression in THP-1 Ctrl and MAVS KO cells. GAPDH was used as a loading control. *C*, IB analysis of endogenous SAMHD1 from the cytosol and mitochondrial fraction of THP-1 Ctrl and MAVS KO mock-infected or infected with SeV for 6 h (MOI of 10). *D*, IB analysis of endogenous SAMHD1 from the cytosol and mitochondrial fraction of HEK293T cells mock-infected or infected with SeV for 6 h (MOI of 1). *C* and *D*, tubulin and VDAC were used as cytosolic and mitochondrial markers, respectively. Equal amounts of protein for both fractions were loaded. *E*, recombinant FL SAMHD1 and MAVS without TM purified from *E. coli* were pulled down with an anti-SAMHD1 antibody and analyzed by IB. *A* and *C*–*E*, representative data from three independent experiments are shown. FL, full-length; hpi, hours postinfection; IB, immunoblot; MAVS, mitochondrial antiviral-signaling protein; MOI, multiplicity of infection; SAMHD, sterile alpha motif and HD domain-containing protein; SeV, Sendai virus; SeV NP, nucleoprotein of SeV; TM, transmembrane; VDAC, voltage-dependent anion channel.

Viral infection of cells activates MAVS (28, 29, 37) and may affect its interactions with other cellular proteins. To examine whether endogenous SAMHD1 and MAVS interact in cells during viral infection, we infected monocytic THP-1 cells with Sendai virus (SeV) for 6 h and then performed IP using SAMHD1 antibodies. We found that endogenous SAMHD1 and MAVS interacted in THP-1 cells regardless of SeV infection (Fig. 2*A*). Of note, THP-1 cells express FL and a shorter splicing variant of SAMHD1 (38) as evident in immunoblot (IB) analysis (Fig. 2*A*). Expression of SeV nucleoprotein (NP) served as a marker of infected cells (Fig. 2*A*).

MAVS specifically localizes to the outer mitochondrial membrane and this localization is required for aggregation of MAVS and subsequent downstream signaling (25, 28, 39). SAMHD1 shuttles between the cytosol and the nucleus (40); however, whether it may also associate with mitochondria has not been investigated. To examine whether SAMHD1 is associated with mitochondria and whether MAVS expression is required for this localization, we performed cytosol and mitochondria fractionation in THP-1 control (Ctrl) and MAVS KO cells. Efficient KO of endogenous MAVS and SAMHD1 expression in these cell lines was confirmed by IB analysis (Fig. 2*B*). THP-1 cells were mock-infected or infected with SeV for 6 h and then subjected to cytosol and mitochondria fractionation and IB analysis. To demonstrate the purity of both fractions, the cytosolic protein tubulin and the mitochondrial protein voltage-dependent anion channel (VDAC) were used as markers (Fig. 2*C*). Interestingly, endogenous SAMHD1 was detected in both the cytosolic and mitochondrial fractions regardless of SeV infection (Fig. 2*C*), indicating that SAMHD1 is associated with mitochondria in THP-1 cells. Moreover, no difference in SAMHD1 association with mitochondria was observed between THP-1 Ctrl and MAVS KO cells (Fig. 2*C*), suggesting that MAVS is not necessary for SAMHD1 association with mitochondria. To examine whether SAMHD1 mitochondrial localization is specific to THP-1 cells only, we also performed the cytosol and mitochondria fractionation using HEK293T cells. Consistently, we found that endogenous SAMHD1 localized in both the cytosol and mitochondria in HEK293T cells regardless of SeV infection (Fig. 2*D*).

To further examine whether SAMHD1 and MAVS directly interact, we performed an *in vitro* pull-down assay using purified, recombinant FL SAMHD1 and MAVS without the TM domain (aa 1–513). Our results demonstrated direct binding of recombinant FL SAMHD1 to MAVS without the TM domain, confirming that the TM domain of MAVS is not required for the binding (Fig. 2*E*). Together, these data suggest that SAMHD1 is a mitochondrion-associated protein likely through its interaction with MAVS.

### SAMHD1 suppresses MAVS aggregation and disrupts IKKε recruitment to MAVS

**Figure 3:**
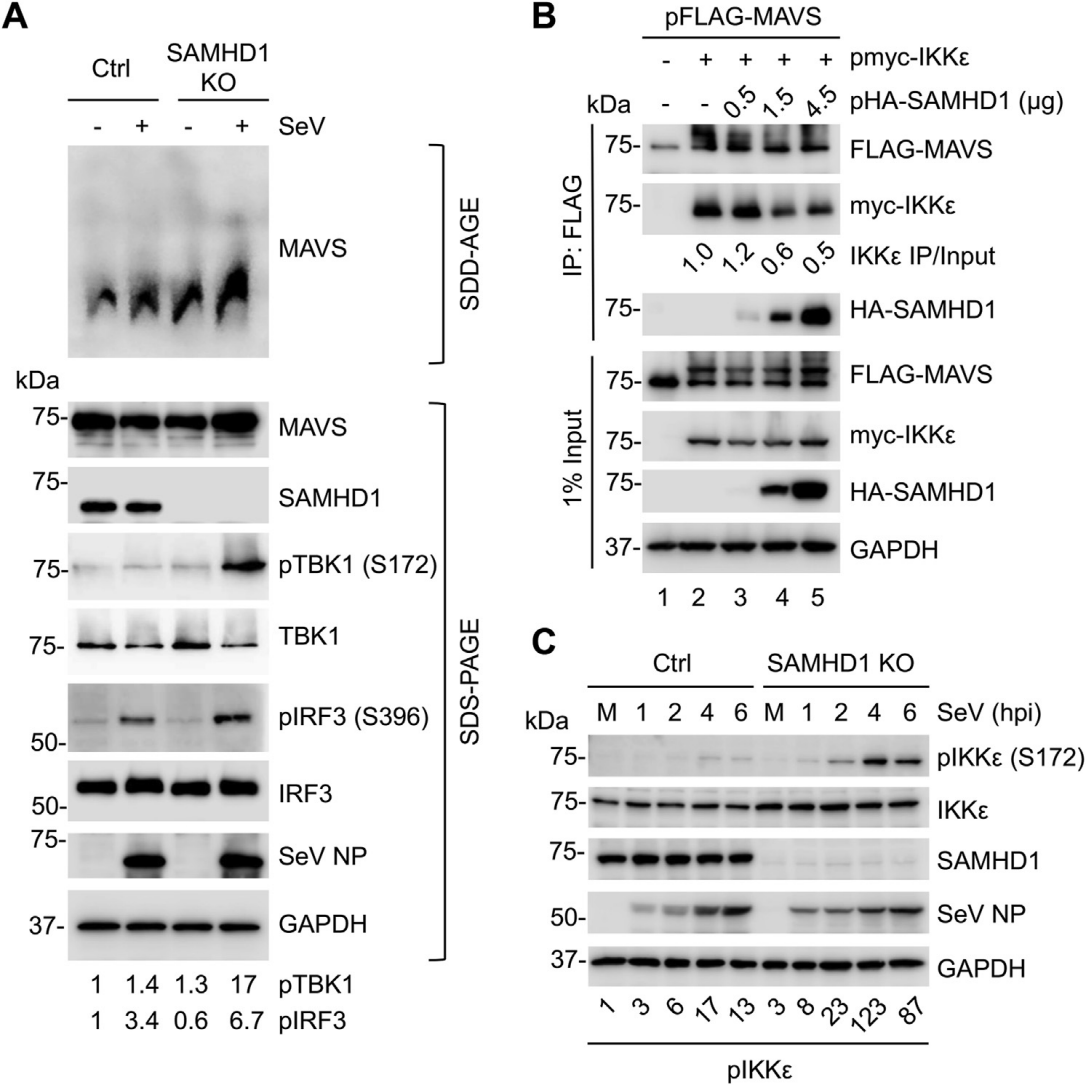
**SAMHD1 suppresses MAVS aggregation and disrupts MAVS interaction with IKKε.***A*, THP-1 Ctrl and SAMHD1 KO cells were mock-infected or infected with SeV (MOI of 10) for 8 h. Crude mitochondria were isolated and subjected to SDD-AGE (*top*). Whole cell lysates were analyzed by SDS-PAGE (*bottom*). Quantification of pTBK1 (S172) and pIRF3 (S396) levels was performed by densitometry and normalized relative to total TBK1 and IRF3, respectively. *B*, HEK293T cells were transfected with expression plasmids encoding the indicated proteins (FLAG-MAVS, myc-IKKε, and increasing amounts of HA-SAMHD1). Cells were lysed 36 h posttransfection, and IP was performed using an anti-FLAG antibody. Indicated proteins were detected by IB. *C*, THP-1 Ctrl and SAMHD1 cells were mock-infected (M) or infected with SeV (MOI of 10) and harvested at the indicated time points. Cell lysates were analyzed by IB with the indicated antibodies. Quantification of pIKKε (S172) levels was performed by densitometry and expressed relative to total IKKε. *A*–*C*, representative results from three independent experiments are shown. IB, immunoblot; IKKε, inhibitor of nuclear factor kappa-B kinase epsilon; IRF, IFN regulatory factor; M, mock infection; MAVS, mitochondrial antiviral-signaling protein; MOI, multiplicity of infection; pIKKε, phospho-IKKε; SAMHD, sterile alpha motif and HD domain-containing protein; SDD-AGE, semidenaturing detergent agarose gel electrophoresis; SeV, Sendai virus; SeV NP, nucleoprotein of SeV; TBK1, TANK binding kinase 1.

We hypothesized that SAMHD1 targets MAVS to regulate the IFN-I signaling pathway. To examine whether SAMHD1 inhibits MAVS aggregation, THP-1 Ctrl and SAMHD1 KO cells were mock-infected or infected with SeV. At 8 h postinfection, crude mitochondria of cells were isolated for analysis. Interestingly, semidenaturing detergent agarose gel electrophoresis (SDD-AGE) analysis showed that SAMHD1 KO THP-1 cells displayed increased MAVS aggregation, and SeV infection further enhanced MAVS aggregation compared to control cells (Fig. 3*A*, SDD-AGE panel). Concomitant to increased MAVS aggregation, SAMHD1 KO THP-1 cells showed a 12-fold and 2-fold increase in phosphorylation of TBK1 (S172) and IRF3 (S396) upon SeV infection, respectively, compared to control cells (Fig. 3*A*, SDS-PAGE panel). Detection of SeV NP served as a marker of virus infection and replication (Fig. 3*A*).

Immediately downstream of MAVS aggregation, the kinases IKKε and TBK1 are recruited to MAVS and activated by phosphorylation (31, 41). To determine whether SAMHD1 can disrupt binding between MAVS and IKKε, Co-IP experiments were performed. FLAG-tagged MAVS was expressed in HEK293T cells alone (Fig. 3*B*, lane 1) or with myc-IKKε (lanes 2–5) in the absence (lane 2) or presence (lanes 3–5) of increasing amounts of HA-SAMHD1. MAVS was immunoprecipitated with an anti-FLAG antibody, and coprecipitation of IKKε was assessed by IB. As expected, IKKε interacted with MAVS, while the amount of coprecipitated IKKε decreased with increasing expression of SAMHD1 (Fig. 3*B*). Previous studies showed that MAVS can be phosphorylated by TBK1 and IKKs (29). Of note, MAVS IB exhibited two bands when IKKε was co-expressed and the upper band could be phosphorylated MAVS (Fig. 3*B*, lanes 2–5 of input samples). Furthermore, upon SeV infection, phospho-IKKε levels were significantly increased (3- to 123-fold) in THP-1 SAMHD1 KO cells compared with control cells (Fig. 3*C*). Thus, SAMHD1 suppresses MAVS activation in response to viral infection, impairs IKKε recruitment to MAVS, and inhibits IKKε phosphorylation upon virus infection.

### SAMHD1 inhibits IKKε-mediated IFN-I signaling and disrupts IRF7 binding to the IKKε kinase domain

**Figure 4:**
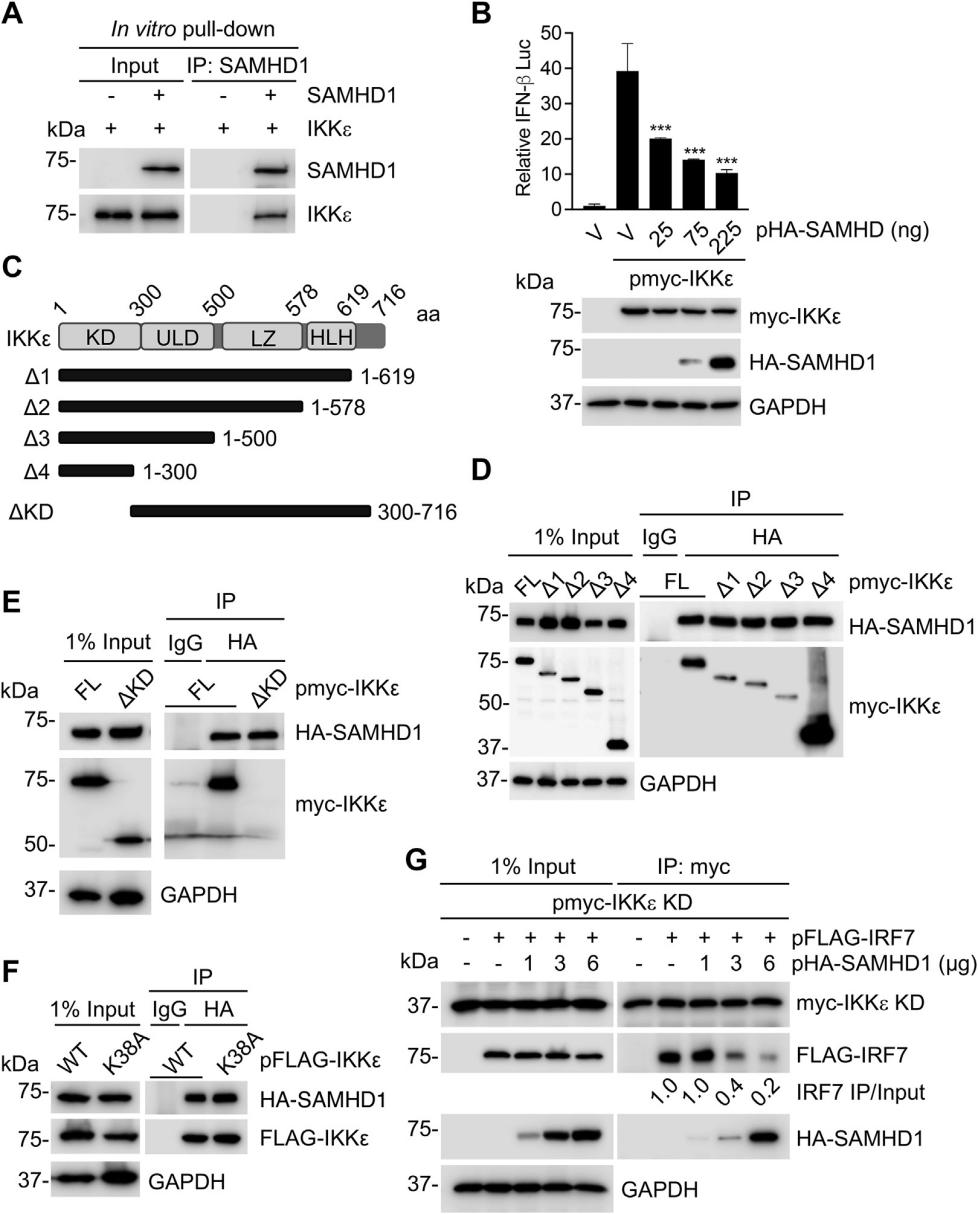
**SAMHD1 inhibits IKKε-mediated IFN-I signaling and disrupts IRF7 binding to the IKKε kinase domain.***A*, recombinant SAMHD1 and IKKε purified from *Escherichia coli* and HEK293T cells, respectively, were pulled down with an anti-SAMHD1 antibody and analyzed by IB. Representative data from two independent experiments are shown. *B*, HEK293T cells were cotransfected with increasing amounts of plasmid encoding HA-SAMHD1, IFN-β-luciferase reporter, renilla-TK, and myc-tagged IKKε. Dual luciferase assay was performed at 24 h posttransfection, and cell lysates were harvested for IB. Error bars represent mean ± SD. Statistical significance was determined using one-way ANOVA; ∗∗∗*p* < 0.001 compared with the vector control in the same group. *C*, schematic representation of full-length (FL) human IKKε (*top*). Five myc-tagged C-terminal and N-terminal deletion mutants are represented by the solid bars below the FL IKKε. The aa numbers of IKKε are shown. *D*–*F*, HEK293T cells were cotransfected with plasmids encoding HA-SAMHD1 and myc-tagged IKKε FL or C-terminal deletion mutants (*D*), IKKε N-terminal deletion lacking the kinase domain (ΔKD) (*E*), or IKKε kinase-inactive mutant K38A (*F*). Cells were lysed, and IP was performed using anti-HA antibody at 36 h posttransfection. Nonspecific IgG was used as a negative control in IP, and the indicated proteins were detected by IB. *G*, HEK293T cells were cotransfected with myc-IKKε KD, FLAG-IRF7, and HA-SAMHD1. Cells were lysed, and IP was performed using anti-myc antibody at 36 h posttransfection. The indicated proteins were detected by IB. *A* and *B* and *D*–*G*, representative data from three independent experiments are shown. HLH, helix-loop-helix domain; IB, immunoblot; IFN, interferon; IKKε, inhibitor of nuclear factor kappa-B kinase epsilon; IRF, IFN regulatory factor; KD, kinase domain; LZ, leucine zipper domain; SAMHD, sterile alpha motif and HD domain-containing protein; ULD, ubiquitin-like domain; V, vector controls; WT, wild-type.

We have previously shown SAMHD1 interacts with IKKε, but not TBK1 (16, 17). However, whether this interaction was direct remained unknown. To address this question, we performed an *in vitro* pull-down assay with purified recombinant FL SAMHD1 and IKKε. Our results indicated direct binding between these two recombinant proteins (Fig. 4*A*). To examine whether SAMHD1 inhibits IFN-I signaling induced by IKKε in cells, we performed an IFN-β promoter luciferase reporter assay. We observed that overexpression of IKKε in HEK293T cells activated the IFN-β promoter luciferase reporter and that co-expression of SAMHD1 inhibited the activation in a dose-dependent manner (Fig. 4*B*).

To map the domains of IKKε responsible for SAMHD1 binding, we constructed a series of truncated mutants of IKKε based on the functional domains of the protein (Fig. 4*C*) and tested their interactions with FL SAMHD1. Myc-tagged FL IKKε and IKKε mutants Δ1-Δ4 (Fig. 4*D*), but not the IKKε mutant lacking the kinase domain (ΔKD) (Fig. 4*E*), coprecipitated with HA-SAMHD1, indicating that the KD of IKKε is required for the interaction. To determine whether IKKε catalytic activity is required for binding to SAMHD1, we used an IKKε mutant (K38A) that ablates its kinase activity (32). We found that the IKKε K38A mutant bound to SAMHD1 similarly to WT IKKε (Fig. 4*F*), suggesting that IKKε catalytic activity is not required for the binding. IKKε amino-terminal KD is sufficient for IRF3/7 binding (42), and we have previously reported that SAMHD1 inhibits IKKε-mediated IRF7 phosphorylation (16). Therefore, we sought to investigate whether SAMHD1 sterically prevents IKKε KD–IRF7 interaction. HEK293T cells were transfected to express myc-tagged IKKε KD, FLAG-IRF7, and increasing amounts of HA-SAMHD1 and then subjected to IP using myc antibodies (Fig. 4*G*). As expected, myc-IKKε KD coprecipitated with FLAG-IRF7. Interestingly, the amount of IRF7 immunoprecipitated with IKKε KD was reduced by SAMHD1 in a dose-dependent manner (Fig. 4*G*). These results suggest that SAMHD1 inhibits IRF7 and IKKε KD interaction, thereby reducing IKKε-mediated IRF7 phosphorylation.

### IRF7-ID is necessary and sufficient for SAMHD1 binding

**Figure 5:**
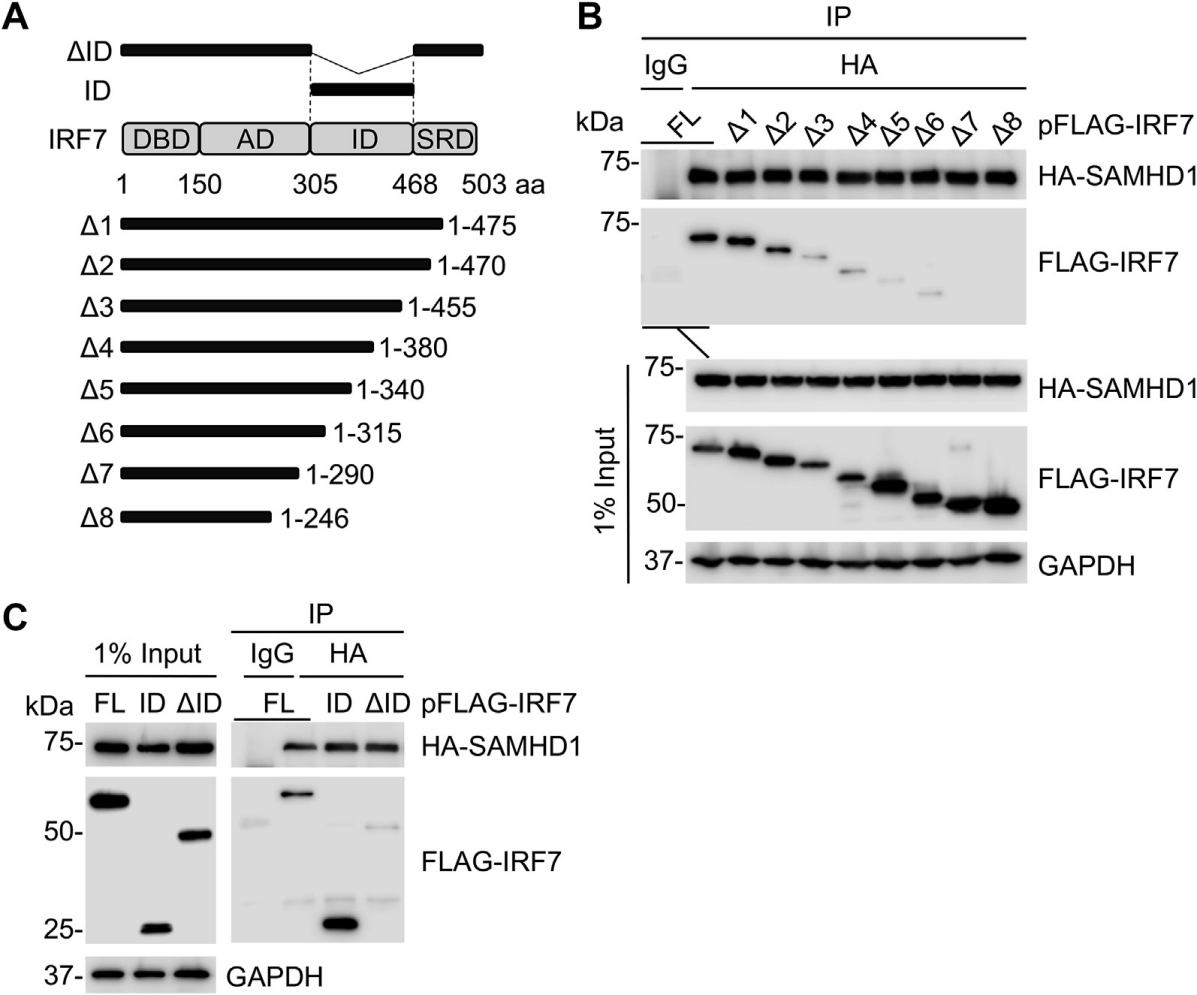
**IRF7 inhibitory domain is necessary and sufficient for SAMHD1 binding.***A*, Human IRF7 is illustrated schematically. The aa numbers of IRF7 are shown. A series of FLAG-tagged C-terminal truncated mutants, ID alone, and ID-deleted mutant (ΔID) are represented by the solid bars. *B*, HEK293T cells were cotransfected with plasmids encoding HA-SAMHD1 and FLAG-tagged IRF7 full length or C-terminal deletion mutant. Cells were lysed and IP was performed using anti-HA antibody at 36 h posttransfection. Nonspecific IgG was used as a negative control in IP, and the indicated proteins were detected by IB. *C*, HEK293T cells were cotransfected with the indicated plasmids. IP was performed as described in (*B*). *B* and *C*, representative results from three independent experiments are shown. AD, activating domain; DBD, DNA-binding domain; IB, immunoblot; ID, inhibitory domain; IRF, IFN regulatory factor; SAMHD, sterile alpha motif and HD domain-containing protein; SRD, serine-rich domain.

Among nine mammalian IRFs identified, IRF3 and IRF7 are the major transcription factors involved in IFN-I induction (43). We have shown that SAMHD1 inhibits IRF7-, but not IRF3-mediated ISRE reporter activation, and IKKε-mediated IRF7 phosphorylation and that SAMHD1 binding to IRF7 is dependent on the histidine-aspartate (HD) domain of SAMHD1 (16). However, the IRF7 domain required for the interaction with SAMHD1 remained unknown. IRF7 functional domains have been extensively studied (44, 45), including the DNA-binding domain, activating domain, ID, and regulatory domain (SRD) (Fig. 5*A*). To identify domains interacting with SAMHD1, we generated several C-terminal truncated mutants (Δ1-Δ7) and performed Co-IP experiments. FLAG-tagged IRF7 FL and truncated mutants were expressed together with HA-SAMHD1 in HEK293T cells. HA-SAMHD1 co-immunoprecipitated with FLAG-IRF7 FL, Δ1, and Δ2, but the interaction was significantly reduced in mutants Δ3-Δ7, suggesting that IRF7-ID is critical for efficient interaction with SAMHD1 (Fig. 5*B*). We then created constructs expressing FLAG-tagged ID alone or FLAG-IRF7 lacking the ID (ΔID). As expected, HA-SAMHD1 co-immunoprecipitated with FLAG-ID but not FLAG-ΔID (Fig. 5*C*). Thus, the ID of IRF7 is necessary and sufficient for its interaction with FL SAMHD1.

### Computational structural prediction of the IRF7-ID and SAMHD1 complex

**Figure 6:**
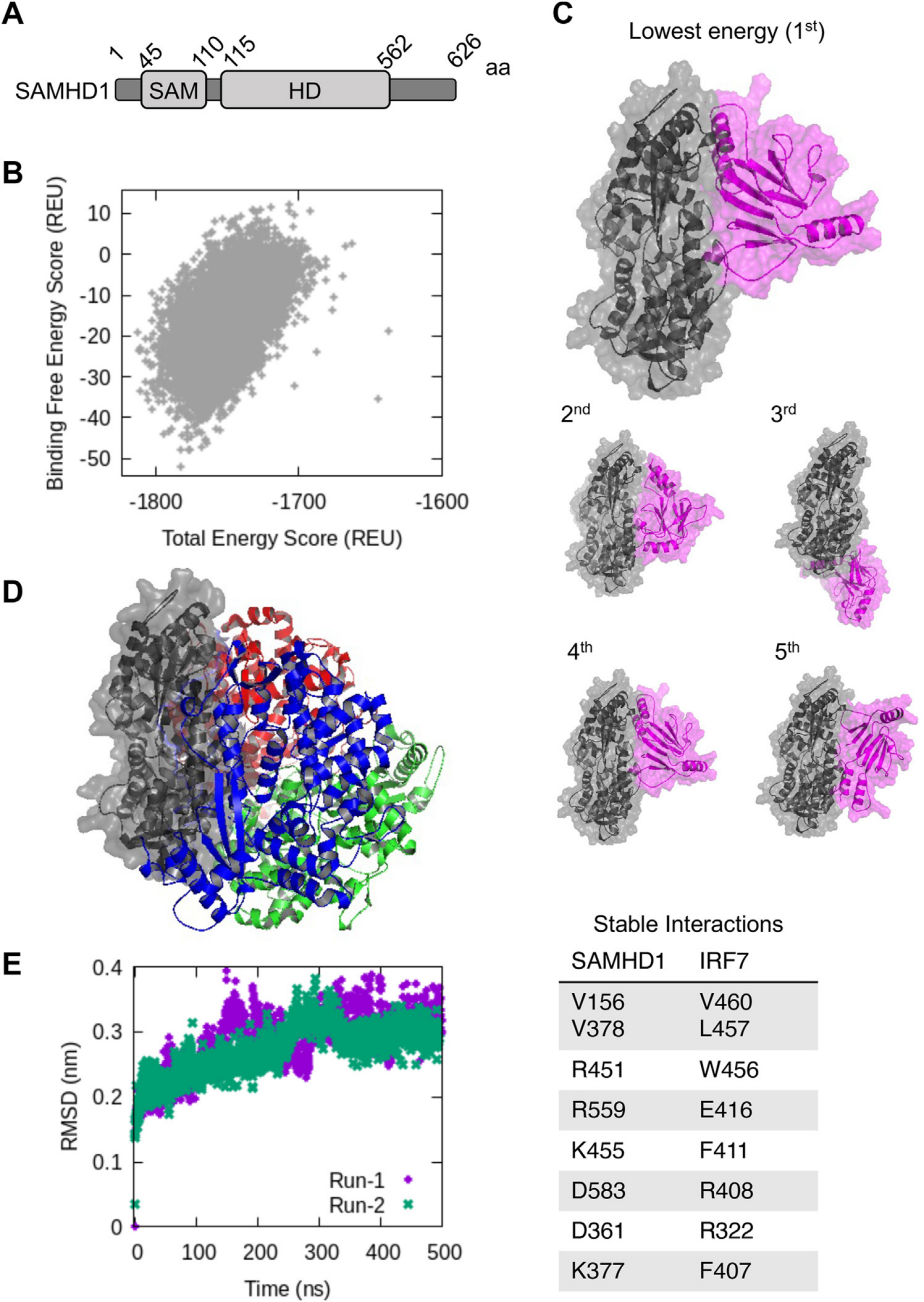
**Computational prediction of IRF7-ID–binding interactions with SAMHD1.***A*, schematic representation of full-length human SAMHD1. The aa numbers are shown. *B*, global docking, showing total energy score *versus* binding free energy score in Rosetta Energy Unit (REU) of 50,000 sampling poses. *C*, the five poses with minimum binding free energy scores. *Gray*, SAMHD1 monomer; *Purple*, IRF7-ID. Note that the fourth best pose is almost identical to the best pose (the first pose), with an RMSD of 1.7 Å. The second and fifth poses have very similar SAMHD1 binding sites with different orientations of IRF7-ID. Only the third best pose has a different SAMHD1 binding within the oligomeric interface. *D*, tetrameric SAMHD1 complex (4bzc in the PDB database) with the same orientation of the *gray* monomer as in (*B*), showing that all best poses in (*B*) bind IRF7 in the oligomeric interface. *Green*, *blue*, and *red* structures are the three other monomers of SAMHD1 in the tetramer. *E*, molecular dynamics simulations of the *top* pose, indicating the stability of the complex at 300 K (*left panel*), along with the observed stabilizing interactions between SAMHD1 and IRF7-ID (Table; note that the first interaction consists of two amino acids from each protein). Amino acid numbers correspond to full-length SAMHD1 and IRF7. ID, inhibitory domain; IRF, IFN regulatory factor; SAMHD, sterile alpha motif and HD domain-containing protein.

To predict binding mode(s) and specific residues in IRF7-ID interacting with SAMHD1, we performed global docking between the two proteins, in which side chain flexibility was sampled in the interaction interface. FL human SAMHD1 monomer (Fig. 6*A*) was taken from its tetrameric complex (36) (pdb id: 4bzc.pdb) and docked on IRF7-ID. A total of 50,000 dockings were attempted, and we sampled 34,935 complex structures that are energy converged (Fig. 6*B*). The best 5 poses, which have the lowest binding free energy scores, all show IRF7-ID binding to the side of SAMHD1 that forms the oligomeric interface in the tetrameric complex (Fig. 6, *C* and *D*). As tetramer formation of SAMHD1 is necessary for its activity (36, 46), these computational results suggest that IRF7-ID not only binds to SAMHD1 but also possibly inhibits SAMHD1 activity.

We also tested the stability of the best predicted complex structure and interrogated its specific intermolecular interactions, at 300 K by employing atomistic molecular dynamics (MD) simulations. Two independent trajectories, each lasting half a microsecond, were generated starting from different random velocities. As seen in Figure 6*E* (left panel), RMSD from the initial structure fluctuated around 0.2 to 0.3 nm in both runs. The complex preserved its structural integrity within the simulation time scale, with the equilibration converging after 0.3 ms. Specific interactions between SAMHD1 and IRF7-ID were also determined. For the top pose, a four-residue hydrophobic cluster between (V156, V378) of SAMHD1 and (V460, L457) of IRF7 as well as salt bridges between D361 of SAMHD1 and R322 of IRF7, D583 of SAMHD1 and R408 of IRF7, and R559 of SAMHD1 and E416 of IRF7 were the primary interactions stabilizing the complex. Additionally, we observed R451, K455, and K377 of SAMHD1 involved in cation-pi interactions with W456, F411, and F407 of IRF7, respectively. The relative stabilities of these important interactions in the binding interface were determined based on distance fluctuations observed in MD and are given in the table in Figure 6*E*, sorted from most to least stable.

### Three key residues of IRF7 important for its transactivation activity and SAMHD1 binding

**Figure 7:**
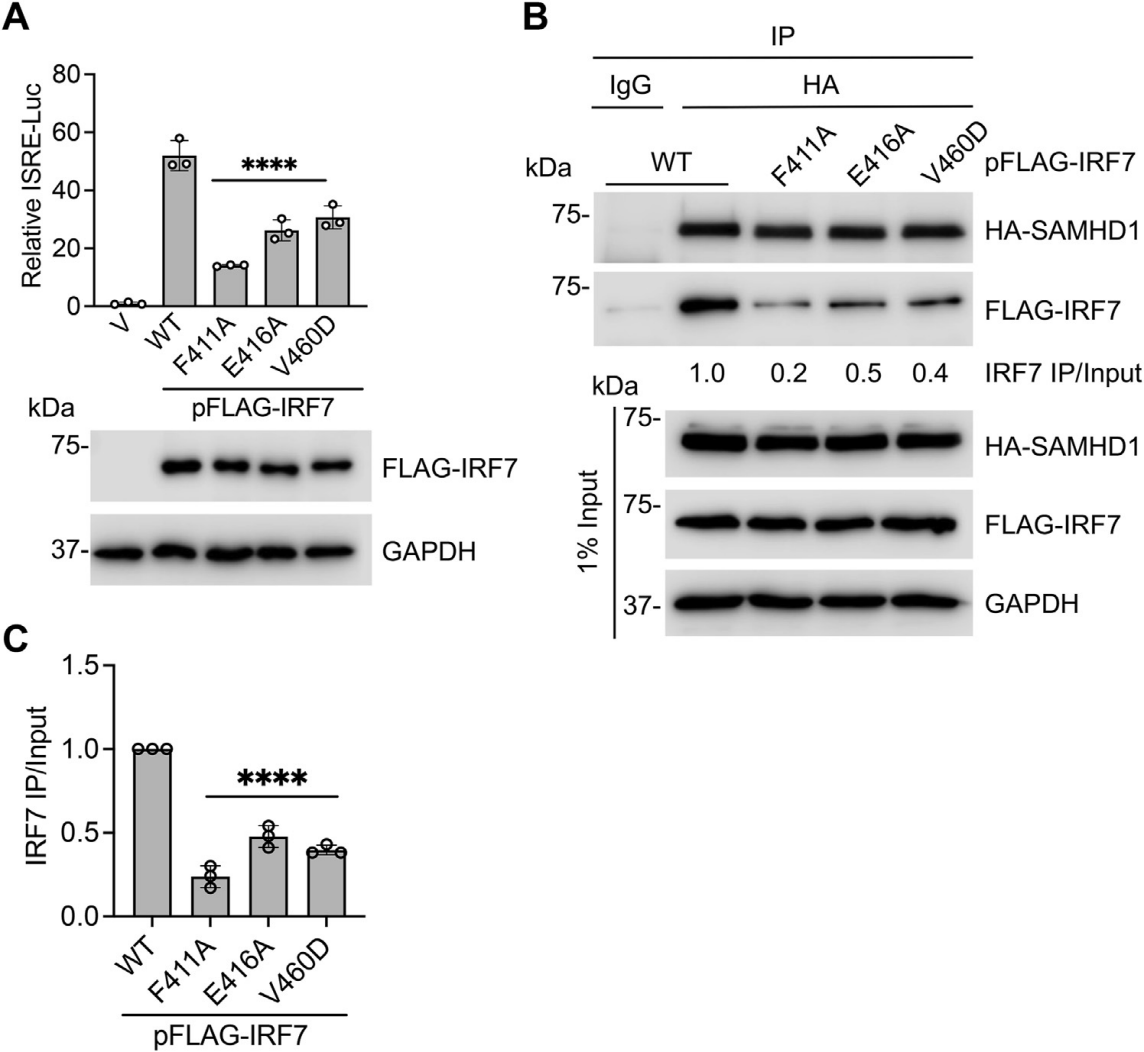
**Three key residues of IRF7 important for its transactivation activity and SAMHD1 binding.***A*, HEK293T cells were cotransfected with ISRE-luciferase reporter, renilla-TK, and FLAG-tagged IRF7 WT or individual mutants as indicated. Dual luciferase assay was performed at 24 h posttransfection, and cell lysates were harvested for IB. Error bars represent mean ± SD. Statistical significance was determined using one-way ANOVA. ∗∗∗∗*p* < 0.0001 compared with IRF7 WT. *B*, HEK293T cells were transfected with expression plasmids encoding HA-SAMHD1 and FLAG-IRF7 WT or individual mutants as indicated. Cells were lysed at 36 h posttransfection, and IP was performed using anti-HA antibody. Indicated proteins were detected by IB. Quantification of IRF7 IP/Input levels was performed by densitometry and expressed relative to IRF7 WT. *C*, average results of relative IRF7 IP/Input levels based on three independent experiments. Error bars represent mean ± SD. Statistical significance was determined using one-way ANOVA. ∗∗∗∗*p* < 0.0001 compared with IRF7 WT. IB, immunoblot; IRF, IFN regulatory factor; ISRE, IFN-sensitive response element; SAMHD, sterile alpha motif and HD domain-containing protein; V, vector control.

To validate the specific interactions between SAMHD1 and IRF7-ID predicted by our MD simulations (Fig. 6*E*), we selected three residues in IRF7-ID expected to destabilize binding interactions for mutagenesis studies. We chose F411A to remove F411-K455 cation-pi interaction, E416A mutation to remove the E416-R559 salt bridge, and V460D mutation to interfere with the hydrophobic cluster at the interface. First, we examined the effect of these mutations on IRF7 transactivation activity in cells using the ISRE reporter assay (44). HEK293T cells were cotransfected to express a luciferase reporter under control of the ISRE promoter and IRF7 WT or individual IRF7-ID mutants (F411A, E416A, or V460D). Interestingly, mutation of these three residues resulted in a significant reduction on IRF7 transactivation activity compared to WT IRF7 (Fig. 7*A*). Next, we evaluated whether these residues are required for IRF7 binding to SAMHD1. IRF7 WT or individual IRF7-ID mutants were overexpressed in HEK293T along with HA-SAMHD1 for Co-IP assays. As expected, HA-SAMHD1 immunoprecipitated with IRF7 WT, while the interaction of the three IRF7 mutants with SAMHD1 was significantly decreased (Fig. 7, *B* and *C*). These results support the computationally predicted binding mode of IRF7-ID to SAMHD1 and demonstrate that residues F411, E416, and V460 in IRF7-ID are important for the transactivation activity of IRF7.

### SAMHD1 binding to IRF7-ID is required for the suppression of IRF7-mediated IFN-I induction

**Figure 8:**
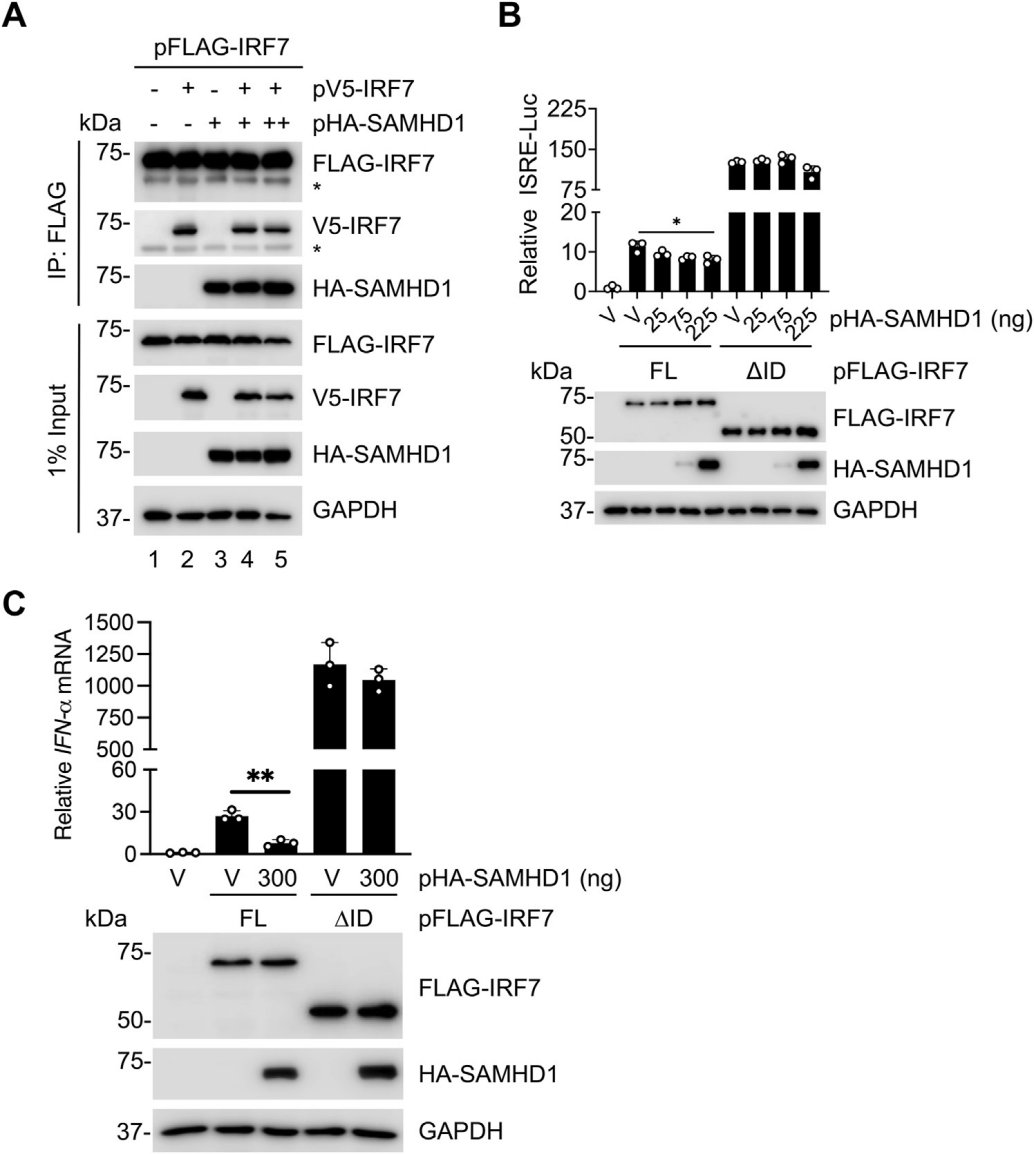
**SAMHD1 binding to IRF7-ID is required for suppression of IRF7-mediated IFN-I induction.***A*, HEK293T cells were transfected with expression plasmids encoding FLAG-IRF7, V5-IRF7, and HA-SAMHD1. IP and IB was performed as described above. (∗) IgG Heavy chain. *B*, HEK293T cells were cotransfected with increasing amounts of plasmid encoding HA-SAMHD1, ISRE-luciferase reporter, renilla-TK, and FLAG-tagged IRF7 FL or ΔID. Dual luciferase assay was performed at 24 h posttransfection, and cell lysates were harvested for IB. Error bars represent mean ± SD. Statistical significance was determined using one-way ANOVA. ∗*p* < 0.05 compared with the vector control in the same group. *C*, HEK293T cells were cotransfected with plasmids encoding FLAG-IRF7 FL or ΔID and HA-SAMHD1. *IFN-α* mRNA levels were measured by RT-qPCR at 24 h posttransfection. *HPRT* mRNA was used as housekeeping control for normalization. Error bars represent mean ± SD. *t* test was used for statistical significance. ∗∗*p* < 0.01. *A*–*C*, representative data from three independent experiments are shown. FL, full-length; HPRT, hypoxanthine phosphoribosyl transferase; IB, immunoblot; ID, inhibitory domain; IFN, interferon; IRF, IFN regulatory factor; ISRE, IFN-sensitive response element; qPCR, quantitative PCR; SAMHD, sterile alpha motif and HD domain-containing protein; V, vector controls.

To evaluate the role of SAMHD1 in the formation of IRF7 homodimer, we tested the effect of SAMHD1 on IRF7/IRF7 interaction. We observed that V5-tagged IRF7 and HA-tagged SAMHD1 interacted with FLAG-IRF7 separately when these proteins were overexpressed in HEK293T cells (Fig. 8*A*, lanes 2 and 3). However, the homodimerization of IRF7 was not significantly altered by SAMHD1 overexpression (Fig. 8*A*, lanes 4–5).

To determine whether interaction between SAMHD1 and IRF7 is required for SAMHD1-mediated inhibition of IRF7-induced IFN-I signaling, we performed the ISRE reporter assay in HEK293T cells. Overexpression of FL IRF7 was sufficient to activate the ISRE promoter, while deletion of the ID (ΔID) resulted in a significant increase in activation as expected (Fig. 8*B*). Interestingly, SAMHD1 overexpression suppressed FL IRF7-, but not ΔID-induced ISRE activation (Fig. 8*B*). Furthermore, SAMHD1 overexpression in HEK293T cells significantly inhibited *IFN-α* mRNA expression induced by IRF7 FL, but not by the IRF7 ΔID mutant (Fig. 8*C*). Together, these results indicate the importance of the SAMHD1 interaction with IRF7-ID for SAMHD1-mediated inhibition of IFN-I induction and signaling.

### Endogenous IRF7 promotes HIV-1 infection and viral transcription in THP-1 cells

**Figure 9:**
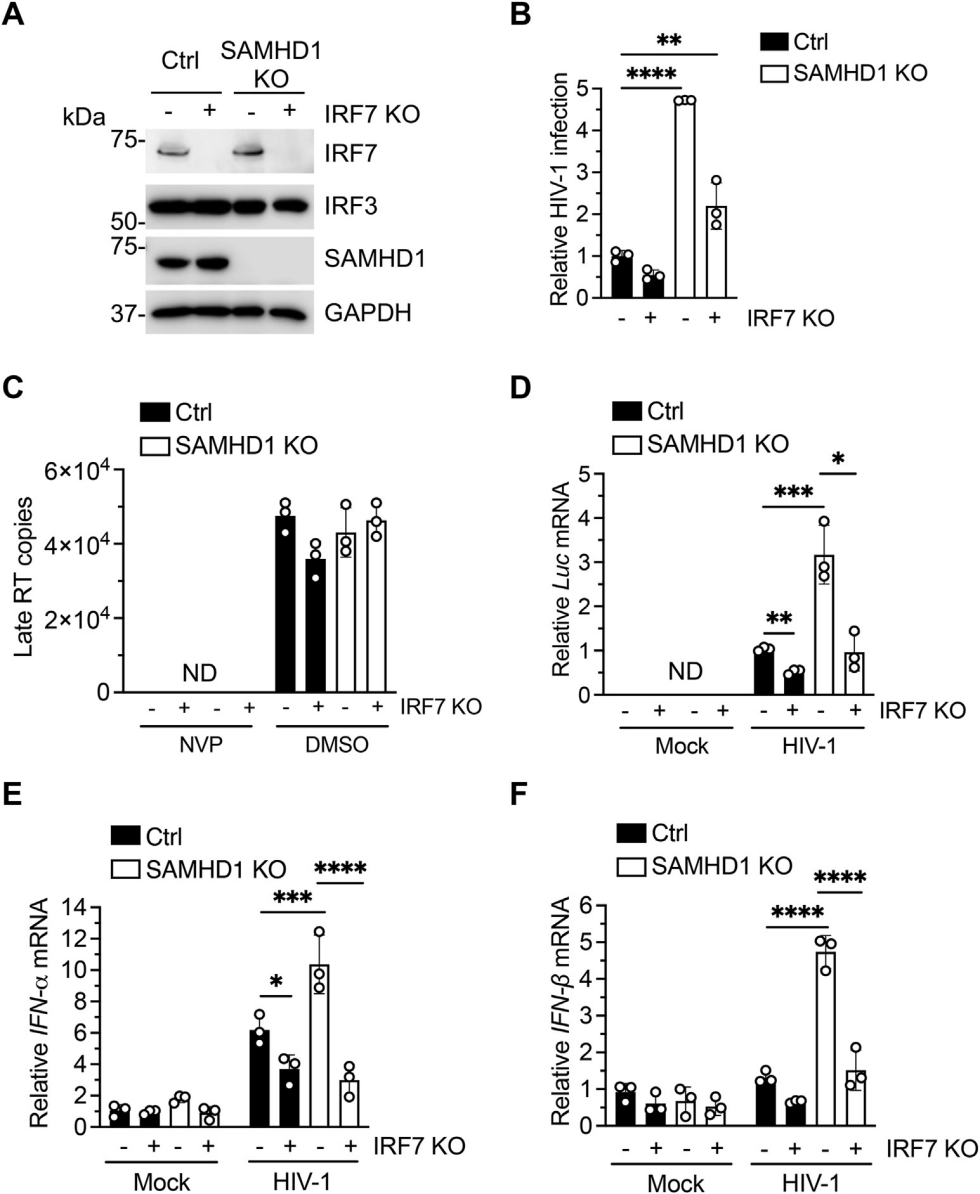
**Endogenous IRF7 promotes HIV-1 infection and viral transcription in THP-1 cells.***A*, immunoblot analysis of IRF7 expression in THP-1 Ctrl and SAMHD1 KO in combination with IRF7 KO. GAPDH was used as a loading control. *B*–*F*, THP-1 Ctrl and SAMHD1 KO in combination with IRF7 KO cells were infected with HIV-1-Luc/VSV-G (MOI of 2). *B*, HIV-1 infection was determined by luciferase assay 24 hpi. Luciferase values were expressed relative to THP-1 Ctrl and normalized to 10 μg of total protein. *C*, genomic DNA was isolated at 14 hpi, and late RT products were quantified by qPCR. Serial dilutions 10^8^ to 10^2^ of an HIV-1 proviral plasmid (pNL4-3) were used to calculate late RT copy numbers. Unspliced GAPDH was used as an endogenous control. *D*–*F*, levels of *Luciferase (Luc)* and *IFN-α/β* (*E* and *F*) mRNA levels were evaluated by RT-qPCR at 18 hpi and 2 hpi, respectively. *HPRT* mRNA was used as a housekeeping control. Values were expressed relative to HIV-1–infected THP-1 Ctrl cells (*D*) or mock-infected THP-1 Ctrl cells (*E* and *F*). *A*–*F*, representative data from three independent experiments are shown. Error bars represent mean ± SD. Statistical significance was determined using two-way ANOVA with multiple comparisons. ∗*p* < 0.05; ∗∗*p* < 0.01; ∗∗∗*p* < 0.001, ∗∗∗∗*p* < 0.0001. HIV, human immunodeficiency virus; hpi, hours postinfection; HPRT, hypoxanthine phosphoribosyl transferase; IFN, interferon; IRF, IFN regulatory factor; MOI, multiplicity of infection; ND, not detectable; NVP, nevirapine; qPCR, quantitative PCR; RT, reverse transcription; SAMHD, sterile alpha motif and HD domain-containing protein; VSV-G, vesicular stomatitis virus G protein.

Induction of IFN-α/β and IFN stimulated genes by HIV-1 in macrophages and resting CD4+ T cells have been documented (47, 48). However, there is evidence for opposing roles of IFN-I on HIV-1 replication and pathogenesis (49, 50, 51). To better understand the role of IRF7 on SAMHD1-mediated suppression of IFN-I signaling during HIV-1 infection, we first examined the effect of IRF7 KO on HIV-1 infection. We generated stable IRF7 KO cell lines in THP-1 Ctrl and SAMHD1 KO cell background. Efficient KO of endogenous IRF7 in these cell lines was confirmed by IB. IRF3 expression was not affected in these cell lines, demonstrating the specificity of IRF7 KO (Fig. 9*A*). We then infected THP-1 Ctrl and SAMHD1 KO cells alone or in combination with IRF7 KO with a vesicular stomatitis virus G protein (VSV-G)–pseudotyped single-cycle HIV-1 expressing a luciferase reporter (HIV-1-Luc/VSV-G) for 24 h. Levels of HIV-1 infection were then measured by luciferase activity in infected cells (52). As expected, SAMHD1 KO resulted in significantly increased HIV-1 infection compared with Ctrl cells. Of note, both THP-1 Ctrl and SAMHD1 KO cells lacking IRF7 expression showed lower levels of HIV-1 infection (Fig. 9*B*), suggesting that IRF7 may promote HIV-1 infection in THP-1 cells.

To determine the stage of HIV-1 life cycle during which IRF7 enhances viral infection, we measured reverse transcription (RT) products using a quantitative PCR (qPCR) assay (18). As a control, treatment with the reverse transcriptase inhibitor nevirapine (NVP) reduced late RT products to undetectable levels (Fig. 9*C*). We observed no difference in the accumulation of late RT products after HIV-1 infection in any of the indicated THP-1 cell lines (Fig. 9*C*), suggesting that IRF7 may enhance HIV-1 infection at a post-RT stage.

To analyze if HIV-1 gene transcription is affected by IRF7 KO, we measured *luciferase (luc)* mRNA expression from HIV-1 genome by RT-qPCR (18). We found that *luc* mRNA was significantly reduced in THP-1 cells deficient for IRF7 expression (Fig. 9*D*). We next evaluated the effect of IRF7 expression on IFN-α/β transcription induced by HIV-1 infection. SAMHD1 KO results in higher *IFN-α/β* mRNA levels when compared with their Ctrl counterparts (Fig. 9, *E* and *F*). Of note, *IFN-α/β* mRNA induction was significantly reduced in THP-1 cells lacking IRF7 expression (Fig. 9, *E* and *F*). These results suggest that endogenous IRF7 in THP-1 cells is important for efficient HIV-1 infection and viral gene expression.

## Discussion

IFN-I is the first line of defense against virus infection. Since excessive immune activation causes detrimental effects on tissue homeostasis, fine-tuning of the IFN-I pathway is of crucial importance. Our group has reported that SAMHD1 inhibits the nuclear factor kappa-B and IFN-I pathways induced by viral infection and pro-inflammatory stimuli (16, 17, 18). Notably, loss of SAMHD1 expression led to reduction of human severe acute respiratory syndrome coronavirus 2 and human coronavirus OC43 replication due to increased IFN-I induction *in vitro*, highlighting the pivotal role of SAMHD1 as a negative regulator of IFN-I signaling (53). Therefore, it is of interest to understand the mechanism by which SAMHD1 exerts its suppression of the IFN-I pathway during virus infection.

Here, we show that SAMHD1 inhibits the activity of the IFN-β promoter induced by the expression of the RLR adapter protein MAVS and IKKε (Figs. 1 and 4). Interestingly, we found that SAMHD1 was associated with mitochondria independently of MAVS expression in THP-1 cells. The CARD of MAVS is essential for MAVS aggregation and subsequent downstream signaling (39). Our finding that SAMHD1 binds to MAVS through the CARD may elucidate the molecular basis for how SAMHD1 suppresses MAVS-mediated antiviral signaling. Consistent with these observations, SAMHD1 KO resulted in enhanced aggregation of MAVS induced by SeV infection (Fig. 3). Upon MAVS activation, several adapter proteins and kinases are recruited to potentiate further antiviral signaling (31, 39, 54, 55). Accordingly, we observed that SAMHD1 disrupted the recruitment of IKKε to MAVS and inhibited TBK1, IKKε, and IRF3 phosphorylation in response to SeV infection. Maintenance of antiviral innate immune homeostasis requires fine-tuning of MAVS-mediated signaling (56, 57, 58), and our results suggest a novel role for SAMHD1 as a negative regulator of MAVS.

SAMHD1 suppressed IFN-I activation induced by IKKε (Fig. 4), and we have shown SAMHD1 inhibited IRF7 phosphorylation by IKKε (16). Mechanistically, our data suggest that SAMHD1 interaction with IKKε contributes to SAMHD1-mediated inhibition of IRF7 phosphorylation by disrupting IRF7 binding to IKKε KD. SAMHD1 binding to IKKε KD may have broader effects, as IRF3 is also recruited to KD for phosphorylation (42). Furthermore, SAMHD1 phosphorylation is a key posttranslational modification that regulates its activity and structure, and four phosphorylation sites have been identified (10, 59). It remains to be determined whether SAMHD1 activity could be modulated by IKKε. Also, IKKε regulates the balance between the IFN-I and type II IFN response by phosphorylating STAT1 and preventing the formation of the gamma-activated factor complex (60). It will be interesting to evaluate whether SAMHD1 suppresses STAT1 phosphorylation by IKKε.

SAMHD1 binds to IRF7 and inhibits IRF7 phosphorylation by IKKε (16). In this study, we showed that SAMHD1 suppression of the IRF7-mediated IFN-I induction was dependent on its interaction with the IRF7-ID (Figs. 5 and 8), and our computational studies predicted the complex structure of IRF7-ID with SAMHD1, providing specific amino acid interactions between SAMHD1 and IRF7-ID (Fig. 6). We also tested the predicted complex using three IRF7-ID mutants (F411A, E416A, and V460D) designed to interrupt some of the binding interactions revealed by MD simulations. We found that all the mutants reduce IRF7 transactivation activity and SAMHD1 binding. In particular, reduced activity of the F411A mutant is a strong indication of the F411-K455 cation-pi interaction predicted by MD because this mutation, unlike the other two, is a substitution within the hydrophobic family. Cation-pi interactions are specific, and therefore such an interaction with positively charged K is restricted to aromatic amino acids. These results demonstrate that IRF7-ID residues F411, E416, and V460 are key residues involved in IRF7 transactivation activity and SAMHD1 binding, consistent with our computational predictions. The IRF7-ID is required for IRF7/IKKε and IRF7/IRF7 interactions, which are essential for IRF7 phosphorylation and dimerization (44, 45), respectively. Therefore, it is possible that mutations of F411, E416, and V460 can also disrupt these interactions. Further structural and functional studies will help determine the mechanisms by which F411A, E416A, and V460D dampen IRF7 transactivation activity.

We did not observe disruption of the homodimerization of IRF7 by SAMHD1; however, SAMHD1 immunoprecipitated with the homodimer IRF7 complex (Fig. 8*A*). It is possible that SAMHD1 may inhibit IRF7 nuclear translocation and/or binding to DNA promoter sequences. Future studies will help determine how SAMHD1 binding to IRF7 suppresses IFN-I activation.

HIV-1 is a weak inducer of the innate immune response (61). However, the interplay between HIV-1 infection and IFN-I induction might be more complex than initially thought. Several IFN stimulated genes target HIV-1 through its life cycle (62, 63, 64) and HIV-1 inhibits IFN-I induction in target cells (65, 66, 67). Yet, IFN-I may contribute to cell activation, which would promote HIV-1 infection and pathogenesis (50, 51, 68). We showed that HIV-1 infection was significantly impaired in cells lacking IRF7 expression. The reduction in HIV-1 infection was accompanied by a decrease in IFN-α/β transcription (Fig. 9). HIV-1 Tat induces transcription from the IRF7 promoter and induces the expression of IFN stimulated genes in the absence of HIV-1 infection, suggesting that HIV-1 may co-opt the function of antiviral host genes for proviral functions (69). Moreover, HIV-1 infection has been shown to stimulate the IRF7-mediated IFN-I response (48), and IRF7 knockdown reduced HIV-1 infection in human primary macrophages (70). A direct correlation between IRF7 expression and HIV-1 latency reactivation in myeloid latently infected cells has also been reported (71), further supporting the positive role of IRF7 on HIV-1 infection and gene expression. Additionally, our molecular docking findings suggest that IRF7-ID binding to SAMHD1 may sterically compete with assembly of SAMHD1 into its tetrameric form. Further functional studies are required to determine how IRF7 promotes HIV-1 infection in monocytic cells.

**Figure 10:**
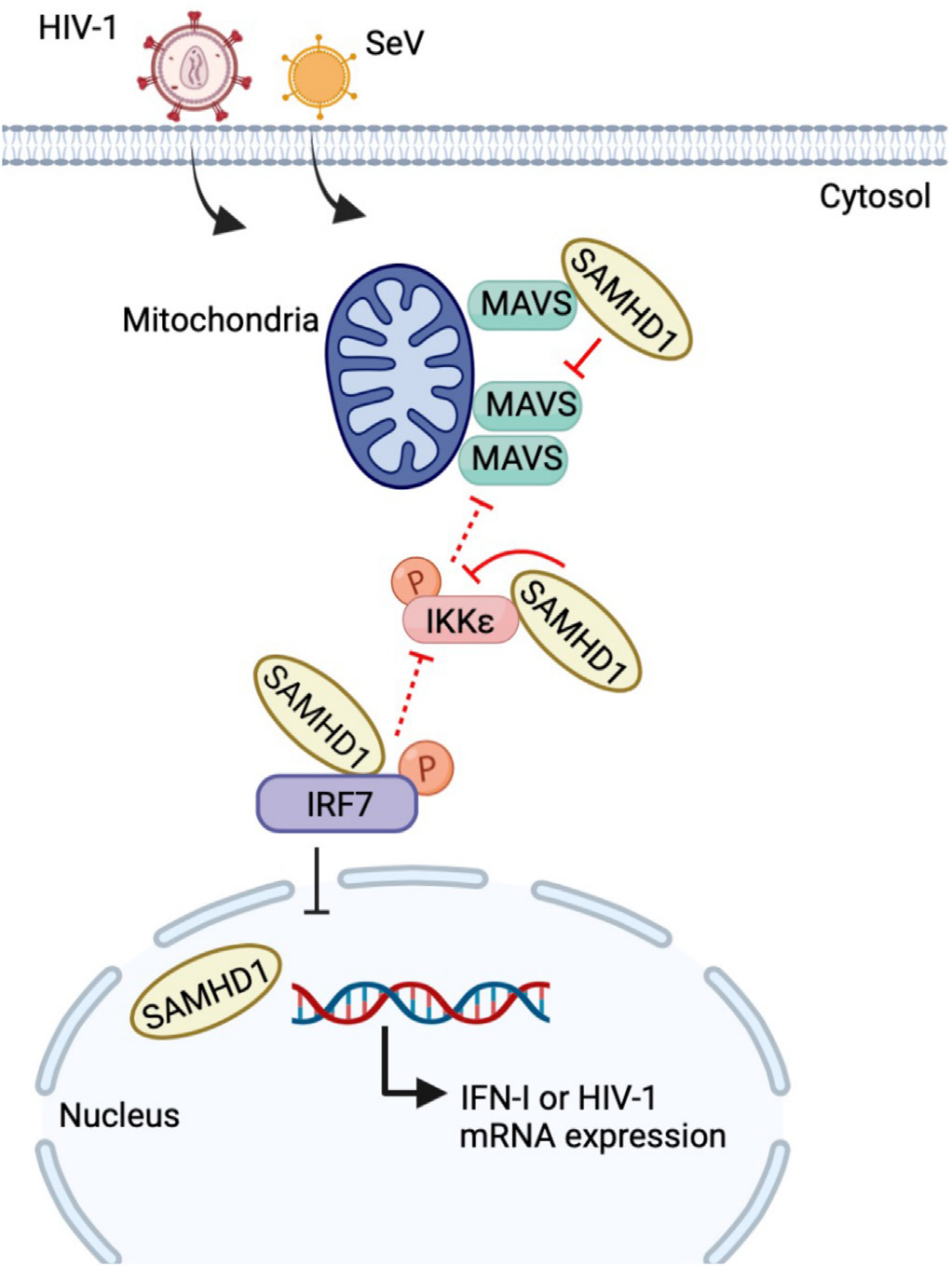
**SAMHD1 impairs IFN-I induction through the MAVS, IKKε, and IRF7 signaling axis during viral infection.** SAMHD1 inhibits MAVS- and IKKε-mediated IFN-I induction in monocytic cells. Mechanistically, SAMHD1 interacts with the CARD of MAVS and suppresses MAVS aggregation, resulting in reduced IKKε recruitment to MAVS and IKKε phosphorylation (indicated with a letter P). SAMHD1 also binds to the kinase domain of IKKε and disrupts binding of IRF7 to IKKε KD. SAMHD1 suppression of the IRF7-mediated antiviral response depends on its interaction with the inhibitory domain of IRF7. Our results also indicated that endogenous IRF7 in THP-1 cells is required for efficient HIV-1 infection and viral gene expression. The *red* T-shaped symbols indicate inhibitory functions confirmed in experiments, while the *black* T-shaped symbols indicate proposed inhibitory mechanisms. This figure was created with BioRender.com. CARD, caspase-recruitment domain; HIV, human immunodeficiency virus; IFN, interferon; IKKε, inhibitor of nuclear factor kappa-B kinase epsilon; IRF, IFN regulatory factor; KD, kinase domain; MAVS, mitochondrial antiviral-signaling protein; SAMHD, sterile alpha motif and HD domain-containing protein.

In summary, our results provide new insight into the mechanisms by which SAMHD1 antagonizes IFN-I induction through the MAVS, IKKε, and IRF7 signaling axis in cells (Fig. 10). Our findings help define the function of SAMHD1 in antiviral innate immune responses, autoimmune diseases, and inflammation.

## Experimental procedures

### Cell culture

THP-1 control and SAMHD1 KO cells have been described previously (72). THP-1 IRF7 KO cells were generated by CRISPR/Cas9 technology. THP-1 control and MAVS KO cells (73) were kindly provided by Dr Greg Towers (University College London). THP-1 cell lines and HEK293T cells were cultured as described (16). GHOST/R5/X4 cell line was cultured as described (74). All cell lines were maintained at 37 °C, 5% CO_2_ and confirmed free from *mycoplasma* contamination using the universal *mycoplasma* detection kit (Abm, G238) following manufacturer’s instructions.

### Virus stocks and viral infection assays

SeV was propagated in specific pathogen-free 10-days embryonated chicken eggs (Charles River Laboratories) and titered on LLCMK2 cells (75) and SeV infection was performed as described (16). Single-cycle HIV-1 luciferase pseudotyped with VSV-G (HIV-1-Luc/VSV-G) was generated in HEK293T cells and titrated in GHOST/R5/X4 cells (16). Virus stock was pretreated with DNAse I (40 U/ml) (Thermo Fisher Scientific) for 1 h at 37 °C. THP-1 cells were infected with HIV-1-Luc/VSV-G at a multiplicity of infection of 2 in the presence of polybrene (10 μg/ml), and viral infection was determined by a luciferase assay (Promega) according to manufacturer’s instructions. Luciferase values were normalized to total protein content of each sample. When indicated, cells were pretreated with NVP (5 μM) for 2 h prior to infection. NVP was maintained in the medium throughout the infection and subsequent culture.

### CRISPR/Cas9-mediated gene editing

THP-1 Control and SAMHD1 KO cells were transduced with two lentivirus vectors encoding for IRF7-targeting guide RNA and CRISPR/Cas9 in the presence of polybrene (10 μg/ml, Sigma). Guide RNA–targeting IRF7 sequences were 5′-GATGCACTCACCTTGCACCG and 5′-GGCAGATCCAGTCCCAACCA. At 48 h posttransduction, cells were maintained in selection media containing blasticidin (10 μg/ml, Gibco). After 5 days, single cell–derived clones were isolated by limiting dilution. The isolated single clonal IRF7 KO cell line was confirmed by immunoblotting.

### Plasmids

The plasmids encoding HA-tagged SAMHD1 WT and HD/RN, FLAG-tagged IRF7, FLAG-tagged IKKε, ISRE-luciferase, and IFNβ-promoter luciferase have been described (16). The C-terminal and N-terminal deletion mutations of MAVS, IKKε, and IRF7 were generated by overlap PCR mutagenesis with Phusion DNA polymerase (New England Biolabs). The plasmid encoding IRF7 ΔID (lacking inhibitory domain) was generated using inFusion mutagenesis kit (Takara) following the manufacturer’s instructions. The plasmids encoding IRF7 F411A, E416A, and V460D were generated using Gibson Assembly master mix (New England Biolabs). Mammalian expression plasmids encoding human FLAG-tagged MAVS, myc-IKKε, and V5-IRF7 were created by insertion of a complementary DNA fragment containing each ORF into the multicloning site of a pcDNA3 (−) (Invitrogen), pCMV4, and pcDNA5/FRT/TO V5 (Addgene plasmid #9081) vectors, respectively. Sequence integrity of newly generated plasmids was confirmed by DNA sequencing. FLAG-tagged kinase-inactive IKKε K38A was a gift from Tom Maniatis (Addgene plasmid #26202). plentiCRISPR v2-Blasticidin was a gift from Mohan Babu (Addgene plasmid # 83480).

### Luciferase reporter assay

HEK293T cells were cotransfected with 50 ng of firefly luciferase reporter plasmids, 5 ng/well of pTK-Renilla, and the indicated amount of expression vectors with PEI. DNA was kept constant during the transfections by the addition of empty vector control plasmid. Transfected cells were harvested in passive lysis buffer (Promega). Luciferase activity was assessed using the Dual-luciferase reporter assay (Promega), following manufacturer’s instruction on VICTOR Nivo Multimode Microplate Reader. The relative stimulation of reporter-gene expression was calculated by normalizing firefly luciferase activity with renilla luciferase activity. In all cases, data shown are representative from at least three independent experiments. Data from experiments performed in triplicate are expressed as mean ± SD.

### RNA isolation and RT-qPCR

Total RNA was isolated using RNeasy Plus kit (Qiagen) following the manufacturer’s instructions. A total of 250 ng was reverse transcribed using iScript cDNA synthesis kit (Bio-Rad). qPCR was performed with iTaq Universal SYBR Green supermix (Bio-Rad) using CFX96 Real Time system (Bio-Rad). Hypoxanthine phosphoribosyl transferase (HPRT) mRNA was used as a control housekeeping gene for normalization. Relative mRNAs expression was calculated by the ΔΔCT method. *IFN-α/β* primers have been described elsewhere (16). Primers used for *HPRT* detection were as follows: forward, 5′- TGACACTGGCAAAACAATGCA; reverse, 5′- GGTCCTTTTCACCAGCAAGCT. Primers for *luciferase* detection were as follows: forward, 5′- GGTTGGCAGAAGCTATGAAACG; reverse, 5′- CATTATAAATGTCGTTCG CGGG.

### HIV-1 late RT product analysis

Cells were harvested and total genomic DNA was isolated using the DNeasy blood and tissue kit (Qiagen) following the manufacturer’s instructions. HIV-1 late RT products were determined as previously described (76). Briefly, 50 ng of DNA was used for iTaq Universal SYBR green (Bio-Rad)-based qPCR detection. Copy number was determined by using pNL4-3 proviral plasmid as a standard. Unspliced GAPDH was used for normalization. Primers of the assay have been previously described (77).

### IB and quantification

Cells were harvested and lysed with cell lysis buffer (Cell Signaling Technology, CST) supplemented with protease and phosphatase inhibitor cocktails (Sigma). Protein concentration was determined by bicinchoninic acid assay (Pierce). Total cell lysates or immunoprecipitates were electrophoretically separated by SDS-PAGE and transferred onto a nitrocellulose membrane (Bio-Rad). Membranes were blocked with 5% nonfat milk and probed with indicated primary antibodies followed by the corresponding secondary antibodies. Primary antibodies used were anti-MAVS (Santa Cruz Biotechnology, sc-166583), anti-FLAG (Sigma, F3165), anti-HA (Sigma, H6908), anti-myc (Sigma, PLA0001), anti-VDAC (CST, D73D12), anti-GAPDH (Bio-Rad, AHP1628), anti-IKKε (CST, D20G4), anti-phospho IKKε (CST, D1B7), anti-TBK1 (CST, 51872S), anti-phospho TBK1 (CST, 5483S), anti-SAMHD1 (Abcam, 67820), anti-SeV NP (MBL, PD029), anti-V5 (CST, D3H8Q), anti-IRF7 (CST, 4920), anti-IRF3 (Abcam, ab50772), anti-phospho IRF3 (CST, D6O1M). IBs were visualized with ECL prime (Amersham) or SuperSignal West Femto (Thermo Fisher Scientific) detection reagents. Images were taken with Odyssey FC (LI-COR). Quantification was performed with ImageJ software (https://imagej.net/ij/index.html) (78).

### Cell fractionation

THP-1 cytosolic and mitochondria fractions were isolated using the Mitochondria/cytosol fractionation kit (Abcam), following the manufacturer’s instructions. In brief, THP-1 cells mock-infected or infected with SeV (multiplicity of infection of 10) for 6 h were washed with cold PBS and lysed by douncing in cytosolic extraction buffer (Abcam). The homogenate was clarified at 1000*g* for 10 min at 4 °C. The supernatant was further centrifuged at 10,000*g* for 30 min at 4 °C to pellet the intact crude mitochondria. Crude mitochondria were resuspended in mitochondria extraction buffer supplemented with DTT and protease inhibitor cocktail. Cytosolic and mitochondria fractions were analyzed by IB.

### Co-IP assay

Cells were lysed in cell lysis buffer (Abcam) supplemented with protease and phosphatase inhibitor cocktails (Sigma). The insoluble fraction was removed by centrifugation at 16,000*g* for 15 min at 4 °C. Complexes were precipitated with Dynabeads Protein G magnetic beads (Invitrogen) and anti-FLAG or anti-HA antibody (Sigma). IgG controls (Sigma, NI03 and NI01) were used when indicated. Beads were washed three times with washing buffer (50 mM Tris (pH7.4), 150 mM NaCl, 1% NP-40, 0.25% sodium deoxycholate) and resuspended in SDS sample buffer (Bio-Rad). Immunoprecipitates were subjected to SDS-PAGE and IB.

### *In vitro* pull-down

The *in vitro* pull-down assay was performed as previously described (16). N-terminal His-tagged FL SAMHD1 was expressed in *Escherichia coli* and purified as described (36). Purified His-tagged MAVS (AR50771PU-N) and c-myc/DDK-tagged IKKε (TP312481) were purchased from Origene.

### SDD-AGE analysis

SDD-AGE analysis was performed as previously described (79). Briefly, harvested cells resuspended in cytosolic extraction buffer (Abcam) were incubated on ice for 10 min and subjected to dounce homogenization. The homogenate was clarified at 1000*g* for 10 min at 4 °C. The supernatant was further centrifuged at 10,000*g* for 30 min at 4 °C to pellet the intact crude mitochondria. Crude mitochondria were resuspended in 6× loading buffer (New England Biolabs) and loaded into a vertical 1% agarose gel (Lonza). Electrophoresis was performed in running buffer (1× TAE and 0.1% SDS) for 40 min with a constant voltage of 100 V at 4 °C. The proteins were transferred to an Immobilon membrane (Millipore) and subjected to IB analysis.

### Molecular docking and MD simulations

The monomeric structure of SAMHD1 was taken from the crystal structure of the tetrameric form (4bzc.pdb, (36)) while that of the IRF7-ID was predicted by Alphafold2 (80). Rosetta 3.12 was employed using its protein-protein docking protocol (81, 82). We performed global docking in a two-stage protocol. In the first stage, large-scale sampling is done by randomizing the relative orientation of the two partners in the centroid mode, allowing all possible translations and rotations. In the second stage, which is a high-resolution phase, smaller movements are done in the full atom resolution followed by energy minimization. The docking protocol assumes fixed backbone while allowing flexible side chain packing. A total of 50,000 attempts were carried out, resulting in 34,935 energy-converged structures. The best poses are selected based on free energy change upon binding, in Rosetta Energy Units.

MD simulations were performed starting from the structure corresponding to the top docking pose. The simulations were carried out using the AMBER99sb-ildn force-field (83), SPCE explicit solvent molecules, and 0.15 M physiological salt concentration (NaCl). Simulation boxes were formed in dodecahedron symmetry in periodic boundary conditions. Particle Mesh Ewald (84) summation method is used for long-range electrostatics. Equilibration phase at T = 300 K includes 10 ns of NVT and 20 ns of NPT simulations. For production level runs, we used 2 fs time steps and NPT ensemble with Parrinello-Rahman barostat (85) at 1 atm. Two independent trajectories were generated, each lasting 0.5 microseconds (totaling 1 μs). All MD simulations were performed by Gromacs package (86) on the UT Southwestern BioHPC supercomputing cluster.

### Statistical analyses

Details concerning the statistical analysis methods and biological replicates are provided in each figure legend. All data were analyzed using GraphPad Prism 9 (https://www.graphpad.com/features) software and were shown as mean and the SD.

## Data availability

All data are contained within the manuscript.

## Conflict of interest

The authors declare that they have no conflicts of interest with the contents of this article.
